# Senescence-related immunosuppressive gene MFAP2 orchestrates stromal-immune crosstalk and immunotherapy resistance in gastric cancer

**DOI:** 10.1186/s12885-026-16660-5

**Published:** 2026-08-04

**Authors:** Yichan Hu, Liaoxiang Zhu, Lijun Zhou, Yichi Zhang, Shunjie Zheng, Jiani Zhu, Ziyu Xi, Zhan Yang, Yifei Hu

**Affiliations:** 1 Lanxi Traditional Chinese Medicine Hospital, Jinhua, Zhejiang 321100 China; 2 Affiliated Jinhua Hospital, Zhejiang University School of Medicine, Jinhua, Zhejiang 321000 P.R. China; 3 Institute of Stomatology, School and Hospital of Stomatology, Wenzhou Medical University, Wenzhou, Zhejiang Province 325000 P.R. China

**Keywords:** Gastric cancer, Senescence-related immunosuppressive gene, Microfibril-associated protein 2, Tumor microenvironment, Tumor-associated Fibroblasts, Immunotherapy

## Abstract

**Background:**

Gastric cancer (GC) is a leading cause of cancer-related mortality worldwide, with poor prognosis and immune checkpoint inhibitor (ICI) resistance mediated by immune escape. Senescence-related immunosuppressive genes (SRIGs) regulate the tumor microenvironment (TME); however, their hub genes and prognostic value in GC remain unclear. This study aimed to identify hub SRIGs and explore their significance.

**Methods:**

Transcriptomic, single-cell RNA sequencing, and spatial transcriptomic data of GC were obtained from The Cancer Genome Atlas and Gene Expression Omnibus databases. Weighted gene co-expression network analysis and Pearson correlation analysis were used to screen for SRIGs. Eight machine learning algorithms identified the hub SRIG, which was validated by RT-qPCR, immunohistochemistry, and clinical samples. Pseudotime trajectory, CellChat, and molecular docking analyses were used to explore the underlying mechanisms and potential drugs. A pan-cancer analysis was used to verify its generalizability.

**Results:**

A senescence-related immunosuppressive signature was constructed, stratifying GC into two subtypes with distinct TME and prognosis. Microfibril-associated protein 2 (MFAP2) was identified as the hub SRIG, significantly upregulated in GC, and an independent prognostic risk factor. It was specifically expressed in extracellular matrix cancer-associated fibroblasts (eCAFs), driving their differentiation and extracellular matrix (ECM) remodeling. MFAP2⁺ eCAFs promoted M2 macrophage polarization via CSF1-CSF1R and C3-C3AR1 axes, mediating immune escape and ICI resistance. AZD4547 and MK-2461 were potential targeted drugs. Pan-cancer analysis confirmed the ubiquitous oncogenic role of MFAP2.

**Conclusions:**

MFAP2 is a hub SRIG linking eCAFs, ECM remodeling, and immune escape in GC. It serves as a reliable prognostic and ICI resistance predictor and potential therapeutic target, providing a basis for precision therapy in GC.

**Supplementary Information:**

The online version contains supplementary material available at https://doi.org/10.1186/s12885-026-16660-5.

## Introduction

Gastric cancer (GC) remains a major global health threat, characterized by persistently high morbidity and mortality rates [1, 2]. Although comprehensive treatment strategies have advanced, patients with advanced disease continue to face challenges, including recurrence, metastasis, and chemoresistance. The median overall survival (OS) often remains below two years, highlighting the urgent need for improved therapeutic outcomes [3, 4]. The emergence of immune checkpoint inhibitors (ICIs) has recently transformed the treatment landscape for advanced GC, providing renewed hope for some patients [5, 6]. However, responses to ICI therapy vary substantially, with only a subset of patients achieving long-term benefits, whereas many experience primary or acquired resistance [7–9]. Therefore, a deeper understanding of the regulatory mechanisms in the GC immune microenvironment and the identification of predictive biomarkers are essential to enable precise patient stratification, overcome resistance, and improve overall treatment efficacy.

Cellular senescence has emerged as a pivotal regulator of the tumor microenvironment (TME), playing a dual role in cancer progression [10–12]. Senescence functions as a tumor-suppressive mechanism by arresting abnormal cell proliferation. Conversely, senescent cells release a spectrum of bioactive molecules known as the senescence-associated secretory phenotype (SASP), which helps shape an immunosuppressive milieu that promotes tumor progression, invasion, metastasis, and therapy resistance [13, 14]. This duality is particularly relevant in GC, where SASP is a key driver of tumor progression and immunosuppression [15]. Nevertheless, the specific functions and regulatory mechanisms of senescence-related immunosuppressive genes (SRIGs) in remodeling the GC microenvironment, mediating immune escape, and contributing to ICI resistance remain poorly understood, representing a significant gap in current research.

The rapid advancement of multi-omics technologies is profoundly transforming cancer research, providing multidimensional insights into tumor biology. However, bulk RNA sequencing remains fundamental for molecular subtyping and biomarker discovery, and single-cell RNA sequencing (scRNA-seq) provides unprecedented resolution in dissecting cellular heterogeneity and subpopulation dynamics [16]. At the spatial level, integrated analytical strategies combining spatial transcriptomics with immunofluorescence co-localization bridge the gap between gene expression and tissue architecture, enabling the visualization of cellular interactions and protein localization in the complex TME [17, 18]. Moreover, machine learning has emerged as a powerful tool for mining high-dimensional biomedical data and identifying core regulatory hubs [19]. This study aimed to integrate these multidimensional technologies to systematically elucidate the role of senescence-related immunosuppressive genes in GC, thereby providing a theoretical and experimental foundation for the discovery of diagnostic biomarkers and therapeutic targets.

In this study, we first identified the senescence-related immunosuppressive phenotype in GC and applied weighted gene co-expression network analysis (WGCNA) and Pearson correlation analysis to identify SRIGs. Based on prognostic SRIGs, distinct molecular subtypes of GC were further characterized, and their correlations with biological functions, immune microenvironment, and patient prognosis were evaluated. Multiple machine learning algorithms were integrated to identify microfibril-associated protein 2 (MFAP2) as the most critical regulator among SRIGs. Single-cell analysis exhibited specific MFAP2 expression in extracellular matrix cancer-associated fibroblasts (eCAFs). Trajectory inference, spatial transcriptomics, and immunofluorescence co-localization were used to map the developmental lineage and spatial niche of MFAP2⁺ eCAFs. Notably, CellChat analysis indicated that these CAFs coordinate an immunosuppressive crosstalk with macrophages, potentially driving M2-like polarization through CSF1-CSF1R and C3-C3AR1 signaling axes.

Furthermore, multi-omics validation confirmed that high MFAP2 expression was a robust predictor of poor prognosis and resistance to ICIs, with a stable predictive performance. Drug-sensitivity screening and pan-cancer analysis further highlighted the therapeutic potential and broad clinical relevance of MFAP2 as a biomarker across multiple malignancies. Consequently, we delineate a key role for MFAP2 in remodeling the immunosuppressive TME and promoting therapy resistance in GC. This study positioned MFAP2 as a promising target to overcome ICI resistance and improve therapeutic precision in GC.

## Materials and methods

### Data download and preparation

Transcriptomic data and corresponding clinicopathological information of patients with GC were retrieved from The Cancer Genome Atlas (TCGA) database (TCGA-STAD, https://portal.gdc.cancer.gov/). Meanwhile, the other three GC datasets were obtained from the Gene Expression Omnibus (GEO) database (GSE29272, GSE15459, and GSE62254, http://www.ncbi.nlm.nih.gov/geo/). When conducting survival analysis, samples without survival information were excluded.

The scRNA-seq or GC data were obtained from the GEO database, including three cohorts (GSE163558, GSE167297, and GSE184198). These datasets contained the gene expression profiles of 14 tumor samples and 6 adjacent normal samples. The R package “Seurat” was used for data preprocessing and quality control, and low-quality cells and potential outliers were removed based on the following filtering criteria: (1) Number of expressed genes < 200 or > 4000; (2) percentage of mitochondrial-encoded genes > 15%; (3) hemoglobin content > 3%. After filtering, the Harmony algorithm was implemented to mitigate potential batch effects between different samples using the R package “Harmony.” Unsupervised cell clustering results were visualized using UMAP plots, and the cell types in each cluster were annotated based on marker genes reported in previous studies.

Spatial transcriptomics data were retrieved from the GEO dataset GSE251950. The pan-cancer immunotherapy cohort and associated clinical information were obtained from the BEST database (https://rookieutopia.hiplot.com.cn/app_direct/BEST/).

### Screening and analysis of SRIGs in GC

The overall transcriptome analysis of GC was based on the TCGA database. Differential analysis with criteria of |logFC| ≥ 0.5 and false discovery rate (FDR) < 0.05 identified 4,644 differentially expressed genes (DEGs). The Tumor Immune Dysfunction and Exclusion (TIDE, http://tide.dfci.harvard.edu/) algorithm was applied to compute the TIDE score, comprising dysfunction and exclusion scores. Additionally, we performed WGCNA on the DEGs to construct a co-expression network and identify modules, thereby screening for key immunosuppressive genes in GC.

Furthermore, to integrate the senescence signatures, gene sets were obtained from the CellAge3 database (https://genomics.senescence.info/cells/*).* We then applied the Pearson correlation coefficient using thresholds of *R* > 0.6 and FDR < 0.001 to select the target SRIGs.

### Functional enrichment analysis

Gene ontology (GO) and Kyoto encyclopedia of genes and genomes (KEGG) enrichment analyses were performed using the R package “clusterProfiler” to elucidate the biological functions and signaling pathways associated with the screened gene sets. To further investigate enriched phenotypes, gene set enrichment analysis (GSEA) was conducted using the R package “GSEA.”

### Unsupervised clustering analysis based on SRIGs to identify GC subtypes

Univariate Cox regression analysis was performed to identify prognosis-related SRIGs in GC. Subsequently, based on the expression of 30 prognosis-related SRIGs, unsupervised clustering of the 370 GC samples was performed using the R package “ConsensusClusterPlus.” The optimal number and stability of GC subtypes were determined using the gap statistic and elbow method. Survival differences among the subtypes were evaluated using Kaplan–Meier (K–M) curves.

### Immunological correlation evaluation

We used multiple computational approaches to comprehensively characterize the tumor immune microenvironment (TIME). Specifically, enrichment scores for immune cell types and oncogenic pathways were computed using single-sample GSEA (ssGSEA). The proportions of 22 tumor-infiltrating immune cell types were estimated using the CIBERSORT algorithm. Additionally, stromal and immune components were evaluated using the ESTIMATE algorithm, yielding immune and stromal scores.

### Machine learning-based screening of the hub SRIG

Eight machine learning algorithms, including AdaBoost, random forest, neural network, multilayer perceptron, k-nearest neighbor (KNN), decision tree, gradient boosting machine, and classification and regression tree, were used to screen for hub SRIG. The dataset was randomly divided into training and independent test sets at an 8:2 ratio with a fixed random seed (42). All models were trained and validated using 5-fold cross-validation exclusively on the training set, while the independent test set was reserved for final evaluation of model performance and was not involved in any training or hyperparameter tuning. Permutation importance was selected as the unified feature ranking metric because it was independent of model architecture, allowing direct comparison of feature contributions across different algorithms. Each feature was permuted 30 times with a fixed random seed to obtain stable estimates. The final key gene was identified as the intersection of the top 10 ranked prognostic SRIGs from each model, as visualized in a petal plot. All computational analyses were performed under Python (version 3.9.7). Key dependencies included scikit-learn (version 1.0.2), TensorFlow (version 2.8.0), and pandas (version 1.3.5). A fixed random seed was set throughout the experiment to ensure reproducibility.

### Differential expression and survival analysis of the hub SRIG

We assessed the expression of the hub SRIG in multiple GC cohorts and evaluated its prognostic value. For survival analysis, patients were stratified into low- and high-expression groups using the optimal cut-off, and K–M curves were generated to compare outcomes between groups.

### Cell-cell communication analysis

Cell-cell communication was analyzed using the R package “CellChat,” which involved constructing a ligand-receptor network to infer communication probability, identifying significant interactions (*P* < 0.05) between two different cell types, and visualizing the strength via chord diagrams and bubble plots.

### Spatial transcriptome analysis

Subsequent spatial transcriptomic analysis was performed on samples GSM7990476 and GSM7990482 from the GSE251950 dataset. The R package “Seurat” was used to integrate scRNA-seq and spatial transcriptome data. The clustering results of single-cell RNA-seq were mapped to spatial transcriptome sections via “FindTransferAnchors” and “TransferData” functions to determine their spatial localization.

### Pseudotime trajectory analysis

Pseudotime trajectory analysis was performed using the R package “monocle” (version 2). Fibroblast subsets of interest were subsetted and a CellDataSet object was constructed. Genes expressed in fewer than 10 cells were filtered out, and those with mean expression ≥ 0.1 were retained for clustering. Dimensionality reduction was performed using tSNE (max_components = 2, num_dim = 6) and DDRTree (max_components = 2, num_dim = 2). DEGs across clusters were identified (qval < 0.01) and used as ordering genes. The DDRTree method was applied for dimensionality reduction, followed by cell ordering with orderCells. MFAP2 expression was mapped onto the reconstructed trajectory, and differences in gene expression across pseudotime stages were compared using one‑way ANOVA.

### Drug sensitivity and molecular docking analysis

To identify potential drugs for GC with high MFAP2 expression, the BEST database was used to correlate MFAP2 expression levels with drug sensitivity data (half maximal inhibitory concentration values) from GDSC and PRISM databases. For molecular docking, candidate drug structures were retrieved from PubChem (https://pubchem.ncbi.nlm.nih.gov/), while the MFAP2 protein structure was predicted according to the AlphaFold database (https://alphafold.ebi.ac.uk/). Protein-ligand docking was performed using AutoDock Vina (version 1.2.2) via the DockEasy website, and the results were visualized using Discovery Studio (version 4.5).

### Molecular dynamics simulation analysis

All molecular dynamics simulations were performed using GROMACS 2022. The receptor was described using the AMBER14SB force field, whereas ligand parameters were generated using Sobtop (version 1.0; dev3.1) based on the GAFF2 force field, with atomic partial charges assigned using the RESP method. The system was solvated in a 1 nm cubic box of TIP3P water, and 0.15 M sodium chloride was added to maintain neutrality. Long-range electrostatic interactions were treated using the PME method with a cutoff of 1.0 nm for non-bonded interactions, and all bonds involving hydrogen atoms were constrained using the LINCS algorithm. Before production, multistage energy minimization was conducted using the steepest descent method, followed by the conjugate gradient method. Simulations were performed under the NPT ensemble at 310 K and 1 bar with a 2 fs time step for 100 ns. Structural stability and conformational changes were analyzed using GROMACS tools: RMSD (gmx rmsd), RMSF (gmx rmsf), hydrogen bonds (gmx hbond) with a donor-acceptor distance cutoff of 0.35 nm and an angle cutoff of 120°, radius of gyration (Rg) (gmx Rg), solvent accessible surface area (SASA) (gmx sasa), and free energy landscape (gmx sham). The binding free energies were calculated using gmx_mmpbsa, with a non-bonded interaction cutoff of 1.0 nm for both electrostatic and van der Waals interactions during energy decomposition analysis.

### Patients and samples

This study protocol was reviewed and approved by the Human Research Ethics Committee of Jinhua Central Hospital, conforming to the Declaration of Helsinki. Patients were recruited from Jinhua Central Hospital, and paired GC and adjacent normal tissues were collected. Written informed consent was obtained from all the participants. The ethical number is 20,262,060,101 (Approval Date: June 18th, 2026). A total of 5 gastric adenocarcinoma patients who received radical gastrectomy between January 2025 and December 2025 were retrospectively enrolled in this cohort. All tissue specimens were reserved for subsequent RT-qPCR, IHC and multiplex immunofluorescence (mIF) detection. Standardized inclusion and exclusion criteria were applied for participant screening: patients with pathologically confirmed primary gastric adenocarcinoma, complete clinicopathological records and intact follow-up data were included, while individuals receiving preoperative anti-tumor therapy, suffering from multiple synchronous malignancies or lacking valid clinical information were excluded. Detailed demographic and clinicopathological features of all enrolled subjects are summarized in Supplementary Table S1.

### Real-time polymerase chain reaction (RT-qPCR) analysis

Total RNA was isolated using TRIzol reagent and treated with DNase I. cDNA was synthesized in a 20 µL reaction volume, following the manufacturer’s instructions. Quantitative real-time PCR was performed using SYBR Green chemistry with gene-specific primers. Relative gene expression was calculated using the 2^–△△Ct^ method and normalized to β-Actin. All samples were analyzed in quintuplicate.

**Table:** 

	Forward: 5’ to 3’		Reverse: 3’ to 5’
MFAP2		GTCCAACAGGAAGTCATCCCAG	CCTGTGTATGGAGTAGAGGCGG
SERPINE1		AGTGGACTTTTCAGAGGTGGA	GCCGTTGAAGTAGAGGGCATT
LBH	GCCCCGACTATCTGAGATCG		GCGGTCAAAATCTGACGGGT
GPX8	TACTTAGGGCTGAAGGAACTGC		GGCTCCGATTCTCCAAACTGA
P4HA3	TCCGAGGATCTTACGGAGAGT		GCCCGGAAATAAGCAAAGGC
β-Actin	CACCTTCTACAATGAGCTGCGTGTG		ATAGCACAGCCTGGATAGCAACGTAC

### Immunohistochemical staining

Paraffin sections were deparaffinized, rehydrated, and subjected to citrate buffer-based antigen retrieval. After endogenous peroxidase blocking and serum blocking, sections were incubated with primary antibodies overnight at 4 ℃, followed by biotinylated secondary antibodies and horseradish peroxidase-streptavidin. 3,3′-Diaminobenzidine was used for visualization, and the sections were counterstained with hematoxylin and imaged.

### Immunofluorescence staining

Paraffin sections were deparaffinized in xylene and rehydrated through a graded ethanol series, followed by rinsing in distilled water. Antigen retrieval was performed by incubation with trypsin at 37 ℃. Sections were then blocked with 5% bovine serum albumin (BSA) at 37 ℃ for 30 min and incubated with primary antibodies overnight at 4 ℃. After washing with PBS, sections were incubated with the appropriate fluorescent secondary antibodies at 37 ℃ for 1 h. Nuclei were counterstained with DAPI, and sections were mounted using an antifade mounting medium. Images were acquired using a confocal fluorescence microscope.

### Cell culture

Primary human dermal fibroblasts (HFF-1 cell line) were obtained from Shanghai Zhong Qiao Xin Zhou Biotechnology Co., Ltd. (ZQ0450) and cultured in DMEM high glucose medium (ZQ-100) supplemented with 15% fetal bovine serum (ZQ0500), 1% penicillin-streptomycin (CSP006), and 1% sodium pyruvate (CSP003). THP-1 cells were obtained from Procell Life Science & Technology Co., Ltd. (CL-0233) and maintained in RPMI-1640 medium (PM150110) supplemented with 10% fetal bovine serum (164210), 0.05 mM β-mercaptoethanol, and 1% penicillin-streptomycin (PB180120). Both cell lines were cultured at 37 °C in a humidified atmosphere containing 5% CO₂. For macrophage differentiation, THP-1 cells were treated with phorbol 12-myristate 13-acetate (PMA) for 24 h to induce differentiation into M0 macrophages, followed by medium replacement.

### Fibroblast transfection and conditioned medium collection

Fibroblasts were transfected with MFAP2-overexpression plasmid or empty vector (VECTOR) control. At 4 h post-transfection, the medium was replaced with fresh culture medium. After 24 h, the conditioned medium was collected and centrifuged at 300 × g for 5 min to remove cellular debris.

### Co-culture and polarization detection

Transfected fibroblasts were harvested for RNA extraction to confirm MFAP2 overexpression efficiency. Conditioned medium from MFAP2-overexpressing fibroblasts or VECTOR control was collected, and M0 macrophages were then incubated with the collected conditioned medium. After 48 h of incubation, macrophages were harvested for RNA extraction and RT-qPCR analysis of M2 polarization markers, including MRC1, CD163, CD68, and IL10, as well as MFAP2.

**Table:** 

	Forward: 5’ to 3’		Reverse: 3’ to 5’
MFAP2		CGCCGTGTGTACGTCATTAAC	CCATCACGCCACATTTGGA
MRC1		GGGTTGCTATCACTCTCTATGC	TTTCTTGTCTGTTGCCGTAGTT
IL-10	GACTTTAAGGGTTACCTGGGTTG		TCACATGCGCCTTGATGTCTG
CD68	CTTCTCTCATTCCCCTATGGACA		GAAGGACACATTGTACTCCACC
CD163	TTTGTCAACTTGAGTCCCTTCAC		TCCCGCTACACTTGTTTTCAC
GAPDH	CGACAGTCAGCCGCATCTT		CCAATACGACCAAATCCGTTG

### Statistical methods

All statistical analyses were performed using R (version 4.2.1), Statistical Package for Social Sciences (version 19.0), Python (version 3.9.7), and GraphPad Prism (version 7.0) software. Measurement data are presented as mean ± standard deviation. Student’s t-test or Mann–Whitney U test was used for comparisons between two groups, while the chi-square (χ^2^) test was applied for comparisons of categorical data. Spearman’s correlation coefficient was used for correlation analysis, and the K–M method with the Log-rank test was used for survival analysis. All statistical tests were two-tailed, and a *P*-value < 0.05 was considered statistically significant.

## Results

### Revealing senescence-related immunosuppressive characteristics in GC through multi-omic integration

**Fig. 1:**
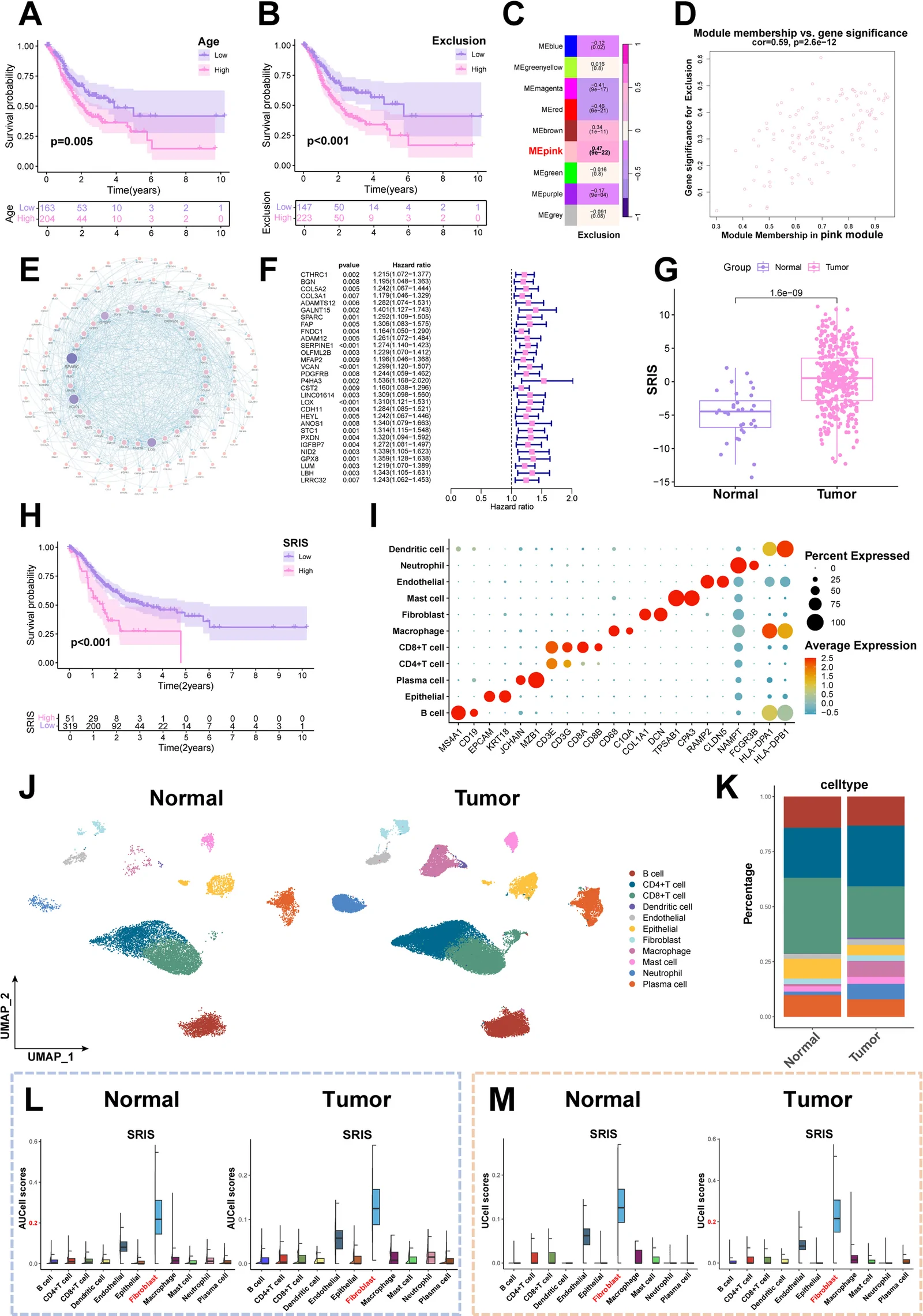
Senescence-related immunosuppressive characteristics in GC through integration of multi-omics. **A** K–M survival analysis of the relationship between age and survival time. **B** K–M survival analysis of the relationship between exclusion score and survival time. **C** Heatmap exhibiting correlations between gene modules and the exclusion phenotype. **D** Scatter plot analysis of genes in the pink module. **E** Co-expression network of senescence genes and immune suppressive genes. **F** Univariate Cox regression of SRIGs. **G** Expression levels of SRIS in normal and tumor tissues. **H** K–M survival curves of patients in high- and low-SRIS groups. **I** Marker gene expression across major cell lineages. **J** UMAP representation of the single-cell landscape across 11 distinct cell clusters. **K** Relative cell proportions in normal and tumor tissues. **L-M** SRIS activity across cell clusters calculated by AUCell and UCell algorithms

Survival analysis indicated that elderly patients with GC had a significantly worse OS compared to younger patients (Fig. 1A). Additionally, a strong tumor exclusion phenotype was significantly correlated with poorer prognosis (Fig. 1B). To identify gene co-expression modules associated with the exclusion trait, WGCNA was performed. Analysis identified the MEpink module exhibited the strongest correlation with the exclusion trait, which was significantly superior to other modules (Fig. 1C). The scatter plot of the MEpink module (Fig. 1D) revealed a significant positive correlation between module membership and gene significance for genes in this module, confirming its centrality to the exclusion phenotype. Consequently, to identify immunosuppressive genes associated with senescence in this key module, we performed Pearson correlation analysis. Figure 1E visualizes these correlations, revealing a co-expression pattern within the “senescence-exclusion” network. This analysis identified 40 senescence-related genes and 95 immunosuppressive genes that were co-expressed; the latter group was therefore identified as SRIGs. From these SRIGs, a subset significantly associated with poor prognosis was further identified through univariate Cox regression analysis (Fig. 1F). This subset was defined as a senescence-related immunosuppressive signature (SRIS), in which all genes were risk factors for adverse outcomes. We further computed SRIS activity score using principal component analysis (PCA) method, revealing its significant upregulation in tumors and association with worse prognosis (Figs. 1G-H).

Collectively, SRIS established in transcriptomic data demonstrated a significant association with GC progression and poor prognosis. To further investigate its role at a higher resolution, we extended our analysis to the single-cell dimension. First, after performing a series of analyses including quality control, batch effect removal, and clustering, the main cell subpopulations in GC were identified based on the previously reported marker genes (B cell, CD4 + T cell, CD8 + T cell, dendritic cell, endothelial, epithelial, fibroblast, macrophage, mast cell, neutrophil, and plasma cell) (Figs. 1I-J and S1) [20]. Figure 1K reveals the proportion differences of each cell population between the normal and tumor group. Using AUCell and UCell algorithms, we evaluated the activity levels of SRIS across various cell types in adjacent normal and GC tissues, revealing a systemic upregulation of SRIS in GC tissues. Notably, fibroblasts exhibited the most pronounced increase in SRIS activity in the TME (Figs. 1L-M). Taken together, these results defined SRIS, which was prognostic, functionally active, and particularly enriched in CAFs.

### Evaluation of TME and immune landscape of SRIG-based subtypes

**Fig. 2:**
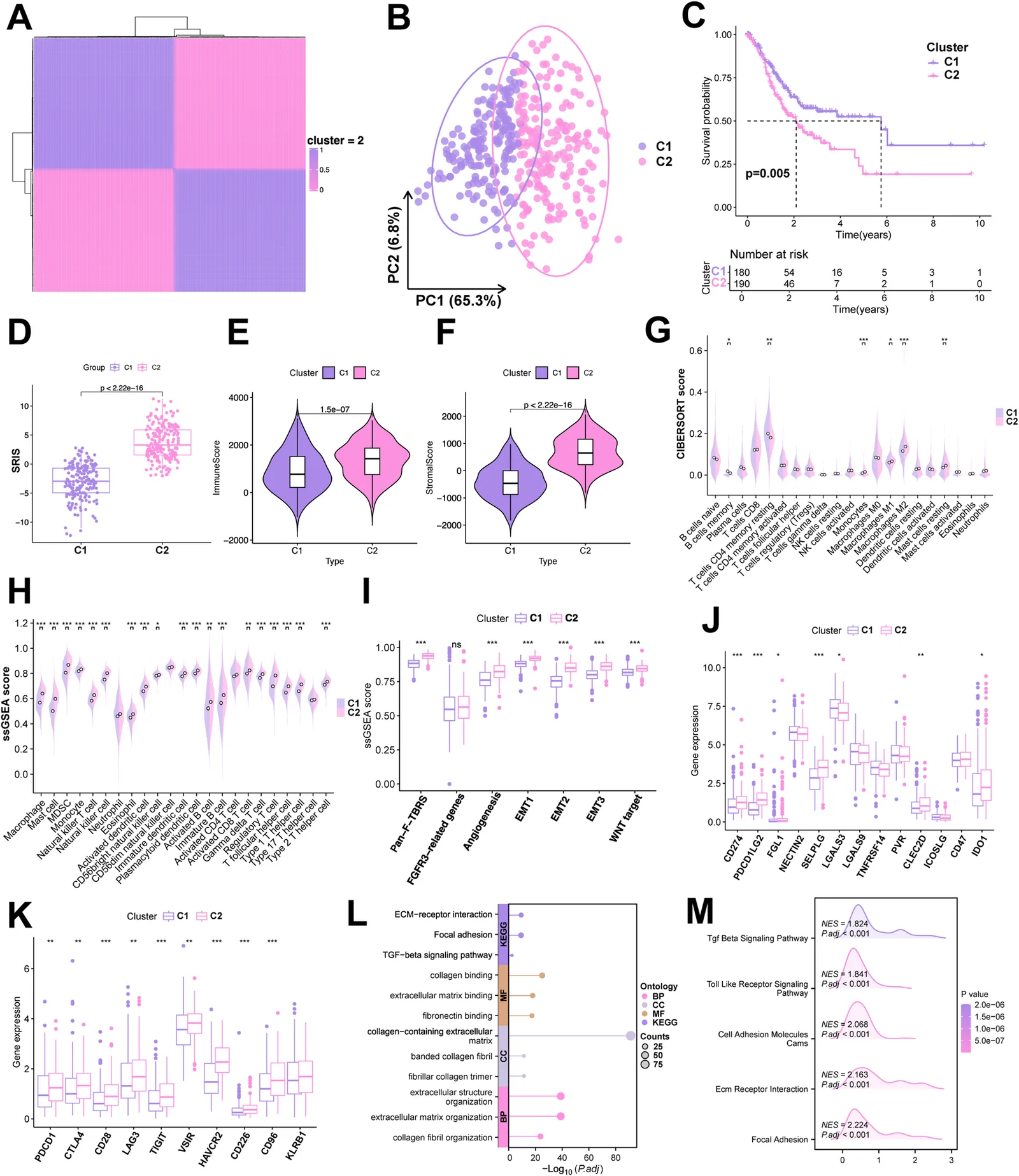
Evaluation of TME and immune landscape of SRIG-based clusters. **A** Unsupervised clustering of prognostic SRIGs and the consensus matrix with k = 2. **B** PCA diagram of clusters C1 and C2. **C** K–M survival curves of patients in clusters C1 and C2. **D** Difference of SRIS score between clusters C1 and C2. **E-F** Differences in immunescore and stromalscore between clusters C1 and C2. **G-H** Differences in CIBERSORT and ssGSEA immune infiltration between clusters C1 and C2. **I** Differences in stromal infiltration between clusters C1 and C2. **J-K** Differences in the expression level of immune checkpoint-related genes between clusters C1 and C2. **L** GO and KEGG enrichment analyses of the differentially expressed genes between clusters C1 and C2. **M** GSEA of biological pathways upregulated in cluster C1

Considering the significance of SRIS in GC, we attempted to elucidate the immunosuppressive state and changes in the TME in GC based on these key genes. Based on SRIS expression, patients with GC were stratified into two distinct clusters (C1 and C2) by unsupervised consensus clustering (Figs. 2 A and S2A–C). PCA confirmed clear separation between clusters, with PC1 explaining 65.3% of variance (Fig. 2B). Figure 2C displays significantly worse OS for cluster C2. Strikingly, cluster C2 also exhibited a markedly elevated SRIS activity score, consistent with its worse prognosis (Fig. 2D). Subsequently, we conducted a detailed comparison of TME composition between C1 and C2. Our analysis revealed that cluster C2 exhibited significantly higher stromal and immune scores compared to cluster C1, suggesting a denser stromal and immune composition in its TME (Figs. 2E-F). Comparative CIBERSORT analysis between clusters C1 and C2 revealed that cluster C2 had significantly higher infiltration levels of several immunosuppressive cell subsets, including M2 macrophages and resting mast cells (Fig. 2G). SsGSEA confirmed and extended this profile, indicating that immunosuppressive infiltration in cluster C2 was much broader, containing Tregs, macrophages, MDSCs, and T-helper 2 cells (Fig. 2H). Additionally, Immune activation-related genes, including CD8A, CXCL10, GZMA, and GZMB, were downregulated in C1 (Fig. S2D). We also calculated the activity scores of stromal-related functions. Cluster C2 exhibited heightened activity in stromal-activation functions, most notably angiogenesis, epithelial-mesenchymal transition (EMT), and extracellular matrix (ECM) remodeling. Meanwhile, stromal-related genes were significantly upregulated in cluster C2 (Figs. 2I and S2E). Moreover, analysis further revealed broad upregulation of most immune checkpoint ligand-receptor molecules in cluster C2, particularly including classic members, including CD274 (PD-L1), PDCD1 (PD-1), and CTLA4 (Figs. 2J-K). Consistently, most genes associated with HLA antigen presentation, CCR chemokine signaling, and CLL-associated lymphocyte regulation exhibited a trend of upregulation in C2 (Figs. S2F–H). Moreover, DEGs between the two clusters were used for functional enrichment analysis. GO and KEGG analyses exhibited significant enrichment in ECM-related functions (Fig. 2L). These findings were consistently reinforced by GSEA, which confirmed the upregulation of these pathways in cluster C2 (Fig. 2M). In summary, the SRIS-high cluster C2 represented an aggressive GC subtype with a convergent phenotype of unfavorable prognosis, intensified immunosuppression, and active stromal remodeling in the TME.

### Identification and validation of MFAP2 as the hub SRIG via machine learning

**Fig. 3:**
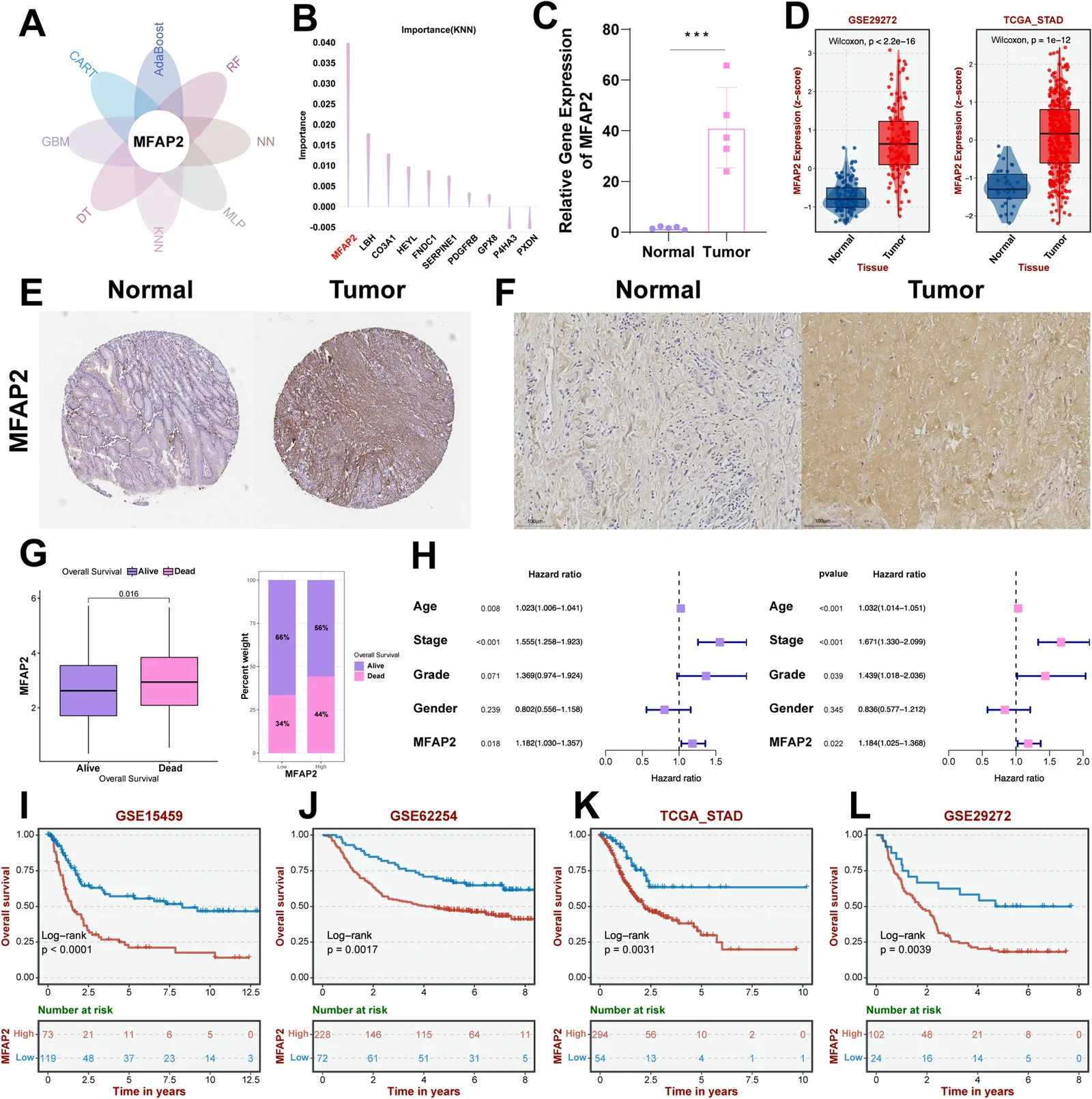
Identification and validation of MFAP2 as the Hub SRIG via machine learning. **A** Petal diagram exhibiting the overlapping gene (MFAP2) based on eight machine learning algorithms. **B** The importance of the top 10 candidate genes within the KNN model. **C** The expression level of MFAP2 detected using RT-qPCR in a clinical sample. **D** The expression level of MFAP2 in tumor and normal tissues from GSE29272 and TCGA cohort. **E-F** Immunohistochemistry images of MFAP2 in normal and tumor tissues from the HPA database and clinical specimens. **G** The expression level of MFAP2 in patients with different survival outcomes. **H** Univariate and multivariate Cox regression analyses of MFAP2 in the TCGA cohort. **I**–**L** K–M curves exhibiting the relationship between MFAP2 expression and survival across four independent cohorts (GSE15459, GSE62254, TCGA-STAD, and GSE29272)

To identify the key prognostic gene from the 30 candidate SRIGs, we integrated a comprehensive machine learning pipeline and q-PCR experiments. Evaluation across the eight algorithms revealed MFAP2 as the consensus top performer and the sole overlap in the top-10 feature listed from all models (Fig. 3A). Among them, the KNN algorithm identified MFAP2 as the gene with the highest score (Fig. 3B). While other top-ranking candidates included P4HA3, SERPINE1, LBH, and GPX8, validation by q-PCR established MFAP2 as the most differentially expressed (Figs. S3A–G). It was upregulated approximately 40-fold in tumors, a significantly greater change than observed for the other four genes (Figs. 3C and S3H–K). These integrated findings established MFAP2 as the prime target for further studies. Its robust upregulation was consistently confirmed across multiple independent cohorts.

In GSE29272 and TCGA-STAD datasets, MFAP2 expression was significantly elevated in tumor tissues compared to normal controls (Fig. 3D). To corroborate our transcriptomic findings at the proteomic level, we first examined protein expression using IHC data from The Human Protein Atlas (HPA) database. HPA data revealed strong MFAP2 protein expression specifically within the tumor stroma of GC, contrasting with weak staining in normal mucosa (Fig. 3E). This stromal-specific pattern was mirrored in our independent clinical IHC samples, which exhibited markedly more intense and abundant MFAP2-positive signals in tumors compared to negligible staining in matched normal tissues (Fig. 3F). Furthermore, we observed that in multiple datasets, MFAP2 expression was steadily elevated in higher-grade or later-stage tumors, associated with more aggressive disease (Figs. S4A-B and S4D). Considering the pronounced upregulation of MFAP2 in GC across multiple levels, we further explored the association between MFAP2 expression and patient prognosis. The results revealed that MFAP2 was an independent risk factor for poor prognosis, as reflected by its higher expression in deceased patients (Fig. 3G) and confirmed by univariate and multivariate Cox regression (Fig. 3H). Furthermore, across multiple datasets, high MFAP2 expression was associated with significantly worse overall, progression-free, disease-free, and disease-specific survival compared to low expression (Figs. 3I–L and S4C). Our data established MFAP2 as a pro-oncogenic factor, a biomarker of poor prognosis, and a potential therapeutic target in GC.

### MFAP2 drove ECM remodeling via fibroblast activation

**Fig. 4:**
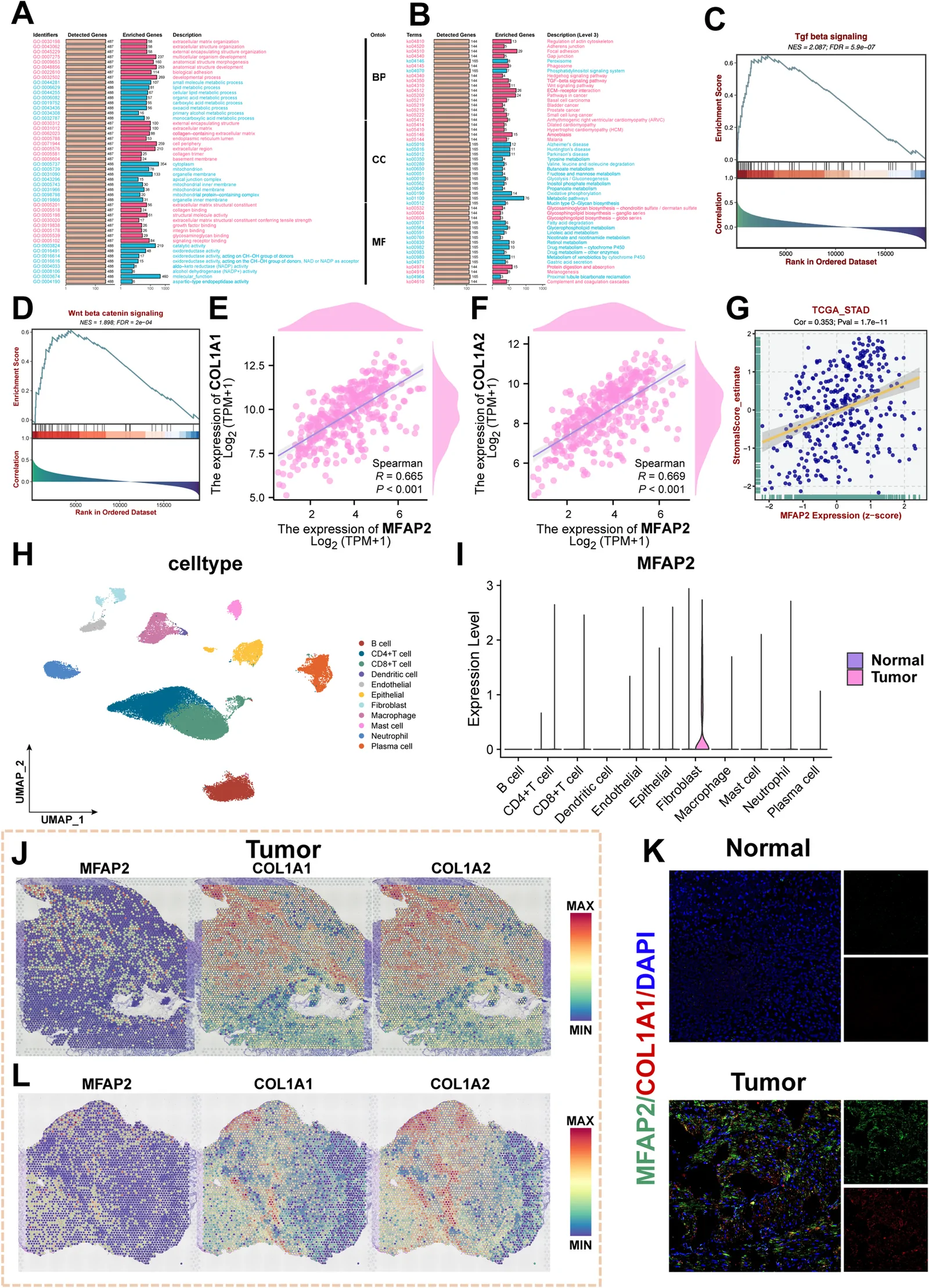
MFAP2 drives ECM remodeling via fibroblast activation. **A-B** GO and KEGG enrichment analyses of MFAP2 co-expressed genes. **C-D** GSEA revealed that TGFβ and Wntβ-catenin signaling pathways were enriched in patients with high expression of MFAP2. **E–G** Correlation analysis between MFAP2 and COL1A1, COL1A2, and stromal score in the TCGA cohort. **H** UMAP plots for cell clustering. **I** Violin plot of MFAP2 expression across all cell types. **J** The spatial arrangement of MFAP2, COL1A1, and COL1A2 expression via spatial transcriptomics analysis. **K** mIF images of the localization and expression of MFAP2 (green) and COL1A1 (red)

To elucidate the functions of MFAP2, we performed GO and KEGG enrichment analyses based on MFAP2 co-expressed genes. GO analysis revealed significant enrichment in ECM and structure organization, localization to the collagen-containing ECM and basement membrane, and molecular functions including ECM structural constitution and collagen binding (Fig. 4A). KEGG analysis further highlighted key oncogenic pathways, including ECM-receptor interaction, focal adhesion, and tumor growth factor-β (TGF-β) signaling pathway (Fig. 4B). GSEA was performed to further define the core biological functions mediated by MFAP2. The significant enrichment of EMT, angiogenesis, coagulation, TGF-β, and Wnt/β-catenin signaling in the high-MFAP2 group (Figs. 4C-D and S4E) suggested that MFAP2 may drive tumor progression through activating these pivotal oncogenic pathways. To delineate the function and cellular context of MFAP2, we performed integrated analyses. MFAP2 expression exhibited significant positive correlations with core ECM and fibroblast markers (COL1A1 and COL1A2) and with a composite StromalScore. This pattern collectively indicated that MFAP2 predominantly functions within the stromal compartment (Figs. 4E–G). ScRNA-seq analysis identified MFAP2 upregulation specifically in fibroblasts of the tumor group (Figs. 4H-I). Further analysis of spatial transcriptome data revealed clear co-localization between MFAP2 and COL1A1/COL1A2 (Figs. 4J and S5A-B). Validation by mIF using clinical specimens revealed that MFAP2 and COL1A1 were markedly overexpressed in GC compared to matched normal tissues and exhibited extensive co-localization (Fig. 4K). Integrated spatial and molecular profiling converged to demonstrate that MFAP2-expressing CAFs were pivotal drivers of ECM remodeling in GC.

### MFAP2⁺ eCAFs mastermind pro-tumorigenic ECM remodeling in GC

**Fig. 5:**
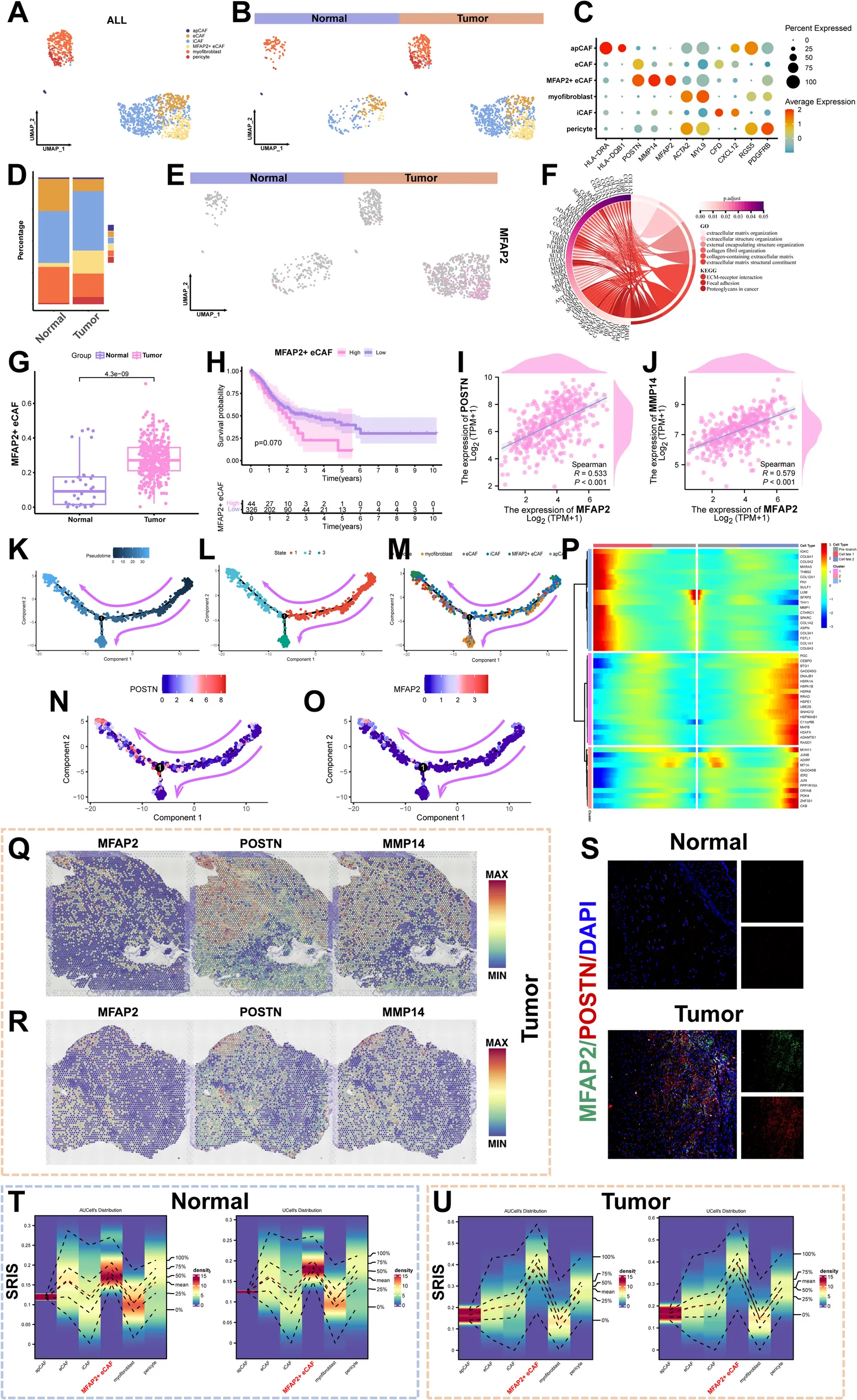
MFAP2⁺ eCAFs mastermind pro-tumorigenic ECM remodeling in GC. **A-B** The UMAP plot of fibroblast subpopulations in normal and tumor groups. **C** Bubble plot displaying the expression of markers across fibroblast subpopulations. **D** Bar plot exhibiting the proportion of fibroblast subpopulations. **E** The UMAP plot depicts the expression level of MFAP2 in the fibroblast subpopulation. **F** GO and KEGG enrichment analyses of the highly expressed genes in MFAP2⁺ eCAFs. **G** Enrichment scores of the MFAP2⁺ eCAFs signature in normal and tumor groups. **H** K–M curve of MFAP2⁺ eCAFs infiltration and OS. **I-J** Scatter plot revealing the correlation between the expression levels of POSTN, MMP14, and MFAP2. **K–M** Developmental trajectory of fibroblast subpopulations. **N-O** Pseudotime projections of POSTN and MFAP2 along the trajectory. **P** Heatmap exhibiting dynamic gene expression during cell differentiation. **Q-R** The spatial arrangement of MFAP2, POSTN, and MMP14 expression via spatial transcriptomics analysis. **S** mIF images of the localization and protein-level co-localization of MFAP2 (green) and POSTN (red). **T-U** SRIS activity across fibroblast subpopulations calculated by AUCell and UCell algorithms

Following the validation of the involvement of MFAP2 in fibroblast pathophysiology, we next used scRNA data to dissect its regulatory effect on fibroblast subpopulations. Using established marker genes, UMAP clustering identified distinct CAF subsets, including eCAFs, antigen-presenting CAFs (apCAFs), inflammatory CAFs (iCAFs), myofibroblasts, and pericytes (Figs. 5A–C and S6A–F) [21]. Notably, we observed a profound pathological expansion of the MFAP2-positive eCAFs subpopulation (MFAP2⁺ eCAFs) in tumor samples compared to normal tissues (Figs. 5D-E). GO and KEGG enrichment analyses of MFAP2⁺ eCAFs further revealed significant enrichment in ECM-related functions (Fig. 5F). To infer the abundance of MFAP2⁺ eCAFs in bulk RNA-seq data, we performed deconvolution analysis using a Bayesian framework, projecting their single-cell-derived signatures onto the TCGA-STAD cohort. The analysis revealed a significant upregulation of MFAP2⁺ eCAFs in GC (Fig. 5G). Patients with higher levels of this subset had numerically inferior survival outcomes, although this association did not reach statistical significance (Fig. 5H). Further supporting its association with the eCAFs phenotype, MFAP2 expression exhibited strong positive correlations with established eCAF markers (POSTN and MMP14) (Figs. 5I-J).

Given its strong association with the eCAFs phenotype, we sought to determine whether high MFAP2 expression is enriched in a specific cell state along the fibroblast differentiation trajectory. Pseudotime trajectory analysis reconstructed a continuous differentiation path for fibroblasts (Fig. 5K), which was segmented into three distinct states (Fig. 5L). Integrating cell subtype annotations revealed that MFAP2⁺ eCAFs mapped predominantly to the terminal pseudotime state, identifying them as a highly differentiated and mature CAF subpopulation in the TME (Fig. 5M). Dynamic expression analysis demonstrated that POSTN and MFAP2 levels increased progressively along the pseudotime axis, reaching their peak during the late-stage maturation of CAFs (Figs. 5N-O). The synchrony of MFAP2 expression with pseudotime progression highlights its pivotal role in MFAP2⁺ eCAFs lineage maturation. To decipher the associated transcriptional reprogramming, we performed BEAM analysis, which identified fate-specific gene modules. A module (Cluster 1) that was robustly activated along the trajectory toward “Cell Fate 1” was notably enriched for ECM structural constituents and pro-tumorigenic regulators. This module included core collagens (COL1A1/A2, COL3A1, and COL6A1), ECM remodeling enzymes (MMP1 and SULF1), and definitive eCAFs markers. The coordinated upregulation of this extensive matrix-production program at the differentiation terminus solidifies the identity of MFAP2⁺ eCAFs as a functionally mature subpopulation committed to constructing a pro-invasive, remodeled microenvironment (Fig. 5L).

Having established MFAP2 as a core component of the MFAP2⁺ eCAFs transcriptional program, we aimed to validate their spatial co-localization in intact tumor tissues. Validation by spatial transcriptomics confirmed that MFAP2, MMP14, and POSTN were co-localized at their transcriptional loci in the tissue architecture (Figs. 5Q-R). Immunofluorescence co-staining further confirmed that MFAP2 and POSTN expression in tumor tissues was significantly increased, and their distribution patterns were extremely similar. Moreover, MFAP2 expression was more intense (Fig. 5S). Moreover, we assessed the SRIS activity across fibroblast subtypes in normal and tumor contexts. While uniformly low in all normal fibroblast subtypes, SRIS was specifically and significantly elevated in MFAP2⁺ eCAFs compared to other fibroblast subtypes within tumors (Figs. 5T-U). Taken together, our integrated research established MFAP2⁺ eCAFs as a functional nexus in the GC microenvironment. They represented a mature cellular state specifically tasked with orchestrating pro-invasive ECM remodeling and were associated with an immunosuppressive senescent landscape.

### MFAP2⁺ eCAFs orchestrated an immunosuppressive microenvironment via targeted cellular crosstalk

**Fig. 6:**
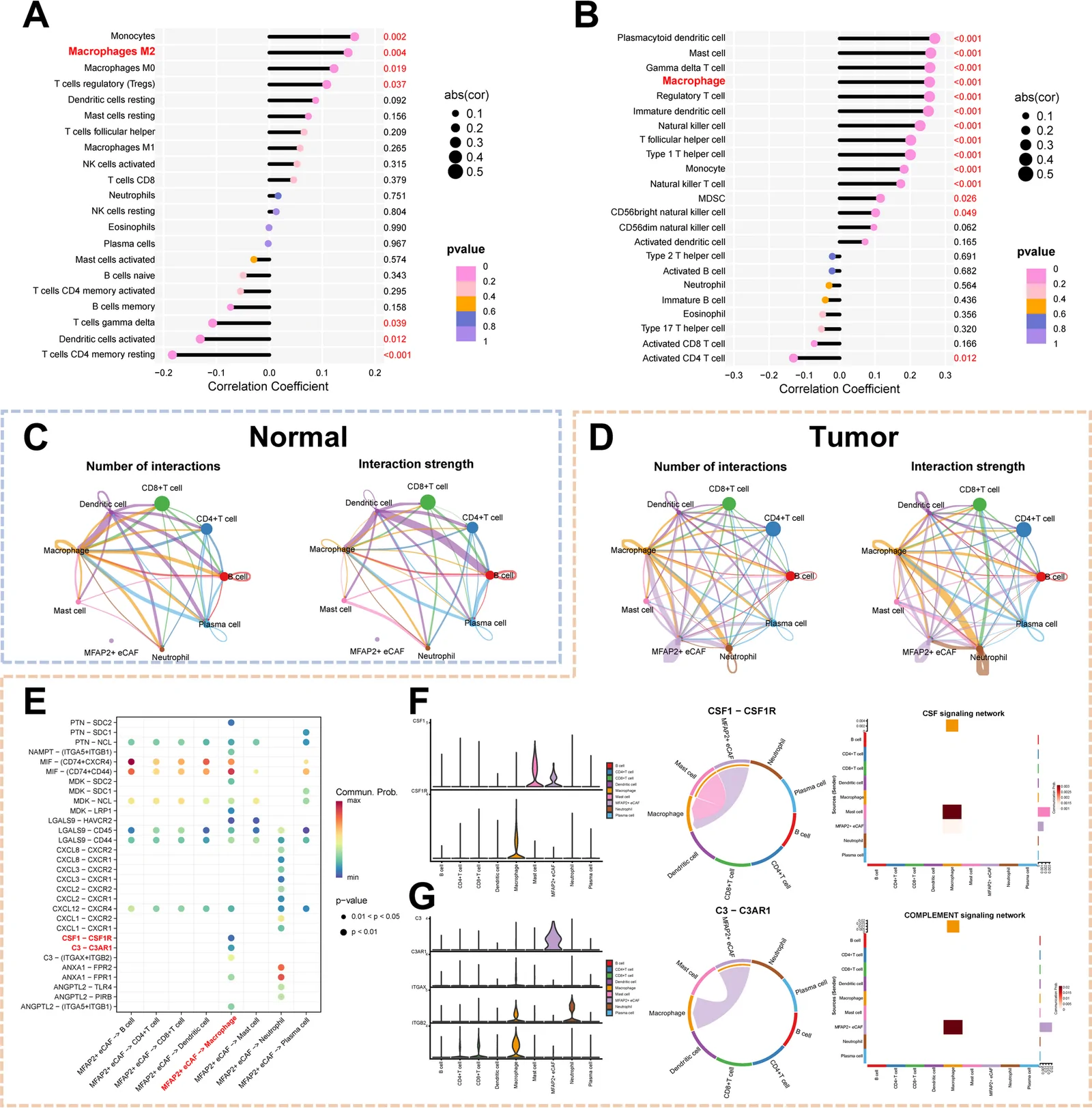
MFAP2⁺ eCAFs orchestrate an immunosuppressive microenvironment via targeted cellular crosstalk. **A-B** Correlation between MFAP2 expression and immune cell infiltration using CIBERSORT (**A**) and ssGSEA (**B**) algorithms. **C-D** Number and strength of ligand-receptor interactions between cell populations in normal and tumor tissues based on CellChat analysis. **E.** Bubble plot of the overall signaling crosstalk between MFAP2 + eCAFs and other immune cells. **F** The expression, signaling network, and overall sender/receiver heatmap of CSF signaling pathway in scRNA data. **G** The expression, signaling network, and overall sender/receiver heatmap of the COMPLEMENT signaling pathway in scRNA data

To delineate the impact of MFAP2⁺ eCAFs on the TIME, we leveraged CIBERSORT and ssGSEA algorithms to assess the association between MFAP2 expression and immune cell infiltration at the bulk tissue transcriptomes. Specifically, MFAP2 expression was positively correlated with immunosuppressive cells (M2 macrophages and Tregs) and negatively correlated with cytotoxic effectors (activated CD4⁺/CD8⁺ T cells) (Figs. 6A-B). These findings indicated that MFAP2 fosters an immunotolerant landscape, characterized by the expansion of suppressive elements and the concomitant exclusion of antitumor immune forces.

To dissect the mechanisms underlying these immune correlations, intercellular communication networks were mapped using CellChat. The analysis revealed that MFAP2⁺ eCAFs underwent a significant functional transformation. From an isolated state with virtually no communication in normal tissue to a central signaling hub that established dense connections with nearly all immune cells in the TME (Figs. 6C-D). Notably, the most prominent signaling flow was from MFAP2⁺ eCAFs to macrophages. This suggested that their pathological activation enables them to recruit and reprogram macrophages through specific ligand-receptor interactions (Fig. 6E). Further analysis of ligand-receptor interactions identified CSF1-CSF1R and C3-C3AR1 axes as specifically mediating the pathway from MFAP2⁺ eCAFs to macrophages. The ligands CSF1 and C3 were overexpressed specifically in MFAP2⁺ eCAFs, while their corresponding receptors, CSF1R and C3AR1, were overexpressed in macrophages (Figs. 6F-G). Notably, CSF1-CSF1R and C3-C3AR1 axes were well-established as canonical signaling pathways that drove macrophage polarization towards an immunosuppressive (M2-like) phenotype [22]. This positioned MFAP2⁺ eCAFs as a source of these key reprogramming signals in the TME.

### Spatial validation of MFAP2⁺ eCAF-macrophage proximity and interaction

**Fig. 7:**
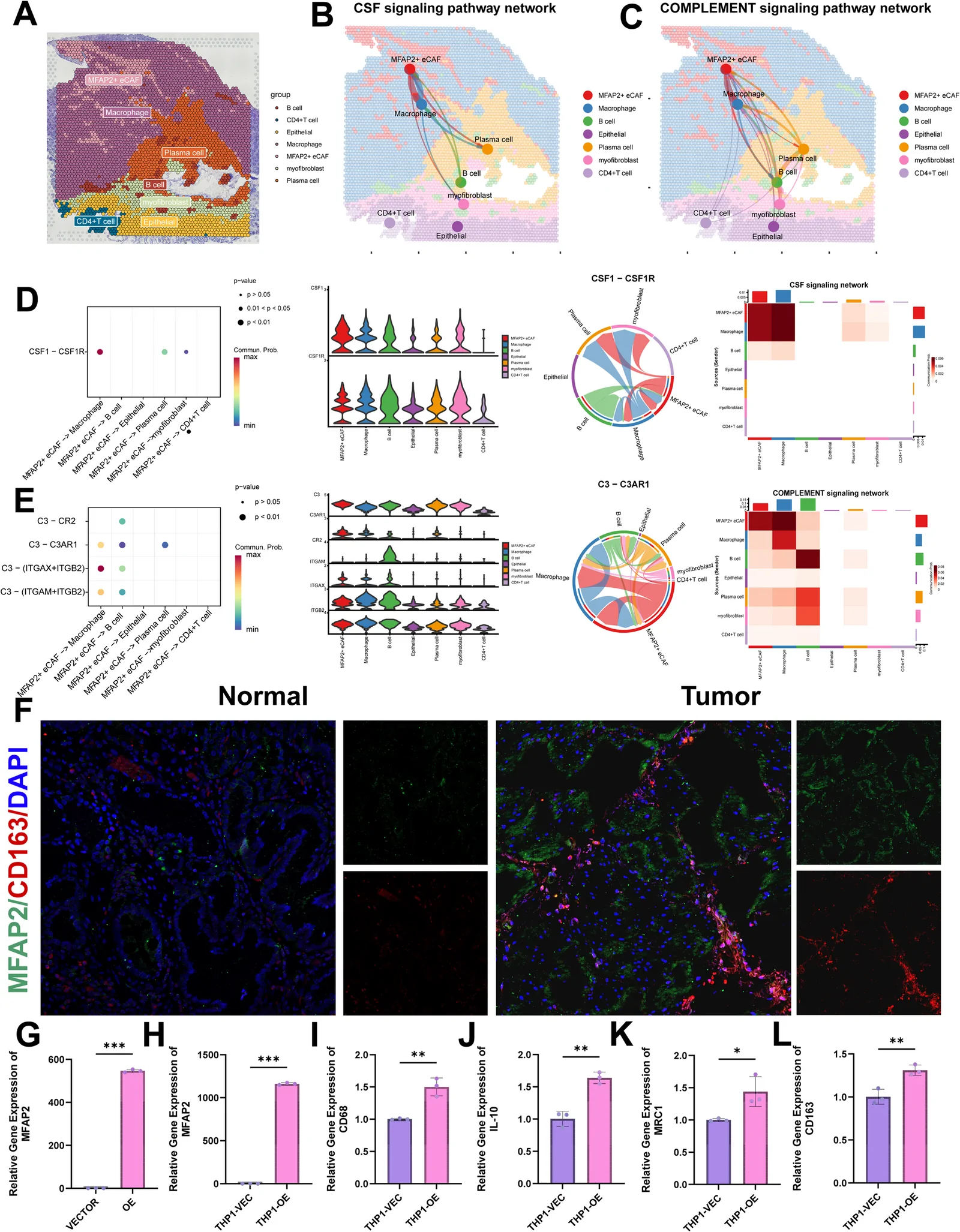
Spatial transcriptomic data validated the proximity and interaction between MFAP2⁺ eCAFs and macrophages. **A** Cell annotation of GC spatial transcriptomic specimens. **B-C** Spatial visualization of CSF and COMPLEMENT signaling pathway networks. **D** The expression, signaling network, and overall sender/receiver heatmap of CSF signaling pathway in spatial transcriptomic data. **E** The expression, signaling network, and overall sender/receiver heatmap of COMPLEMENT signaling pathway in spatial transcriptomic data. **F** mIF images of the localization and protein-level co-localization of MFAP2 (green) and CD163 (red). **G**. RT-qPCR detection of MFAP2 in MFAP2-overexpressed fibroblast cell line (OE) and empty vector control fibroblasts (VEC). **H-L** RT-qPCR detection of MFAP2 (**H**), CD68 (**I**), IL-10 (**J**), MRC1 (**K**), and CD163 (**L**) in THP-1 macrophages after co-culture with OE or VEC fibroblasts

To spatially resolve the predicted crosstalk, we used spatial transcriptomics to map the distribution of MFAP2⁺ eCAFs and macrophages in intact tumor tissue. Their spatial mapping revealed significant co-localization within stromal regions (Fig. 7A). This direct spatial proximity thereby provided the architectural foundation for paracrine signaling between these populations in the TME. We further projected the identified signaling pathways onto spatial coordinates to evaluate their in situ activity. CellChat analysis confirmed that CSF and COMPLEMENT pathways were highly active in regions where MFAP2⁺ eCAFs and macrophages co-existed (Figs. 7B-C). We then examined the spatial expression of the key molecules. High expression of ligands (CSF1 and C3) in MFAP2⁺ eCAFs niches and receptors (CSF1R and C3AR1) in adjacent macrophage zones was evident (Figs. 7D-E). These spatial findings collectively validated the predicted MFAP2⁺ eCAFs-to-macrophage signaling axis and confirmed the alignment between single-cell inference and tissue architecture. Moreover, mIF staining provided definitive histological evidence for the predicted cellular crosstalk. In tumor tissues, we visualized the direct spatial adjacency between the abundantly present MFAP2⁺ eCAFs and CD163⁺ M2 macrophages, which sharply contrasted with their scarce and non-proximal distribution in normal tissues (Fig. 7F). To functionally verify the regulatory effect of MFAP2⁺ eCAFs on macrophage polarization, we established an MFAP2 overexpression (OE) model in THP-1-derived macrophages for in vitro validation. qRT-PCR results demonstrated robust upregulation of MFAP2 transcripts in the MFAP2-OE group relative to the VECTOR control (Fig. 7G-H). Upon MFAP2 OE, THP-1 macrophages exhibited markedly elevated mRNA levels of pan-macrophage marker CD68, anti-inflammatory cytokine IL-10, and classical M2 macrophage specific markers MRC1 and CD163, whereas the baseline expression of these genes remained low in VECTOR-treated macrophages (Fig. 7I-L). These spatial, histological and in vitro transcriptional results together implied that MFAP2 from eCAFs might promote the formation of immunosuppressive M2-like macrophages within the tumor stroma.

### MFAP2 as a predictor of immunotherapy resistance and a potential therapeutic target

**Fig. 8:**
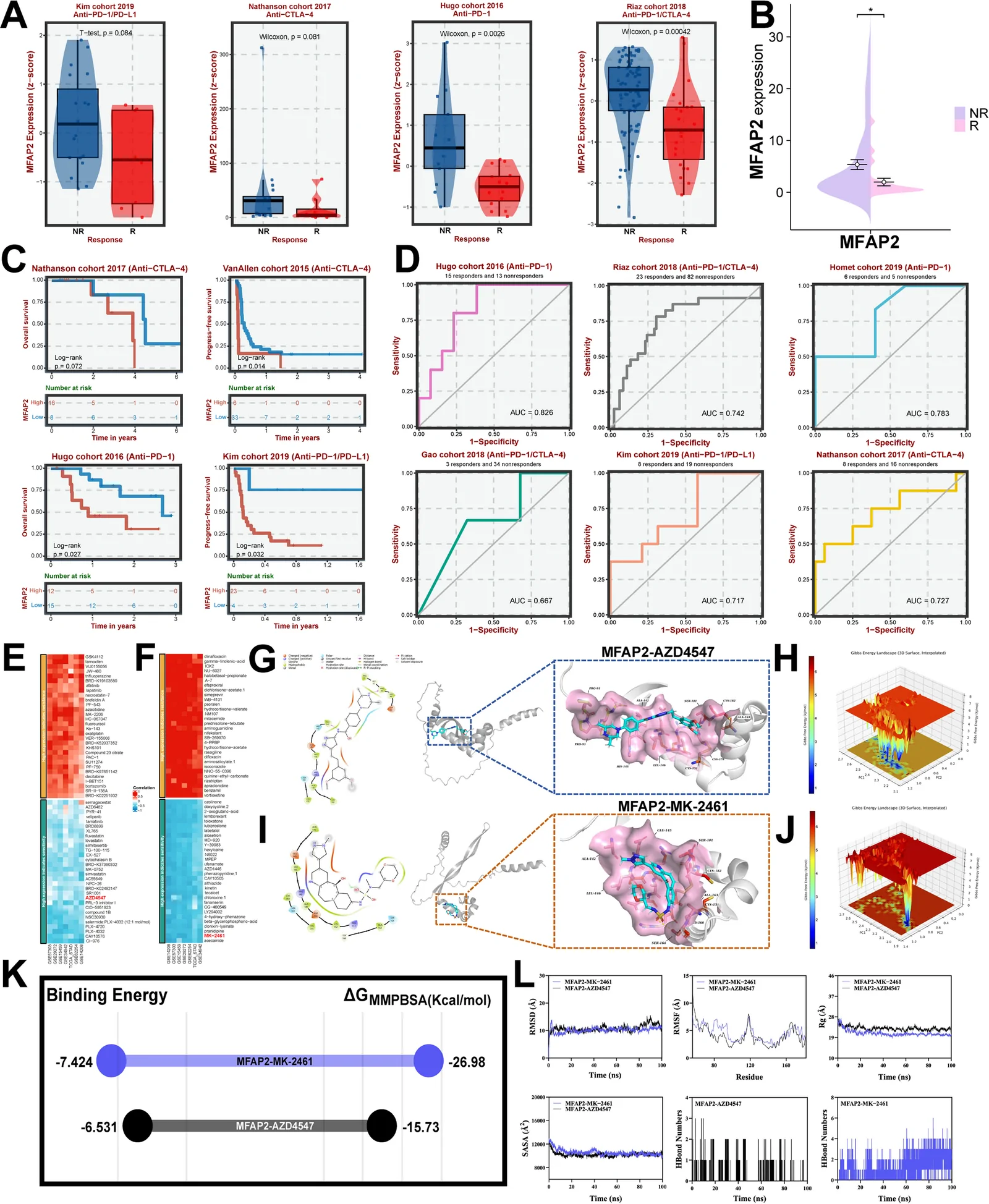
MFAP2 as a predictor of immunotherapy resistance and a potential therapeutic target. **A** Expression of MFAP2 in non-responders (NR) versus responders (R) across Kim, Nathanson, Hugo, and Riaz immunotherapy cohorts. **B** Expression of MFAP2 in NR versus responders R across the PRJEB25780 cohort. **C** K–M curves exhibiting the relationship between MFAP2 expression and survival in immunotherapy cohorts. **D** ROC curves for predicting response rates to immunotherapy based on MFAP2 expression levels. **E-F** Heatmaps revealing the correlation between MFAP2 expression and predicted sensitivity across CTRP and PRISM datasets. **G**,** I** Molecular docking illustrating the binding interactions and key amino acid residues between MFAP2 and its potential inhibitors, AZD4547 and MK-2461. **H **and** J** Gibbs Energy landscape diagrams present the stability of MFAP2-ligand complexes. **K** Bar chart compares the binding energies and Gibbs free energy of MFAP2-ligand complexes. **L** Line plots present dynamic simulation metrics (RMSD, RMSF, hydrogen bond numbers, SASA, and Rg) over time

Guided by our mechanistic insights, we assessed MFAP2’s role in clinical resistance and its potential as a therapeutic target to overcome immune evasion in GC. Analysis across multiple independent pan-cancer immunotherapy cohorts revealed that high MFAP2 expression robustly correlated with resistance to anti-PD-1, anti-PD-L1, or anti-CTLA-4 therapy, with significantly elevated levels in non-responders versus responders (Figs. 8A). This predictive value held true in a dedicated GC cohort, where MFAP2 was similarly upregulated in patients failing to respond to immunotherapy (Fig. 8B). Consistently, high MFAP2 expression correlated with inferior survival outcomes, exhibiting trends of reduced OS and progression-free survival (PFS) in patients across independent immunotherapy cohorts (Fig. 8C). Furthermore, MFAP2 demonstrated robust predictive performance for immunotherapy response across diverse cancer cohorts (Fig. 8D).

Next, we examined the correlation between MFAP2 expression and drug responsiveness, leveraging data from two drug sensitivity databases (CTRP and PRISM). Notably, the analysis highlighted several top candidates, including AZD4547 and MK-2461, which are currently in clinical trials for GC and exhibit predicted sensitivity in MFAP2 high-expression samples (Figs. 8E-F) [23, 24]. Molecular docking and molecular dynamics simulations were performed to explore the potential binding interactions between the identified drugs and the MFAP2 protein. Figure 8K illustrates the initial binding energy and MM/PBSA-calculated binding free energy (ΔG) of MFAP2 with MK-2461 and AZD4547. Molecular docking revealed that AZD4547 and MK-2461 bound to the hydrophobic pocket of MFAP2 with favorable binding affinities. The side chains of AZD4547 (–6.531 kcal/mol) formed a hydrogen bond with SER A:181 and hydrophobic interactions with PRO A:91, PRO A:93, ALA A:142, and LEU A:146. In comparison, MK-2461 (–7.424 kcal/mol) formed hydrogen bonds with GLU A:145, SER A:181, and TYR A:160, alongside hydrophobic interactions with ALA A:142, LEU A:146, and ALA A:163 (Figs. 8G and I). Molecular dynamics simulations were conducted to assess the MFAP2 stability in complex with MK-2461 and AZD4547. Both complexes reached conformational equilibrium within 20 ns, as evidenced by converged RMSD values, consistent radius of gyration (Rg) indicating maintained structural compactness, and stable SASA profiles reflecting preserved protein topology. RMSF analysis confirmed comparable residue flexibility, whereas hydrogen bond monitoring revealed more persistent and abundant hydrogen bonding networks in the MFAP2-MK-2461 complex (Fig. 8L). The analysis of the free energy landscape, utilizing principal components derived from RMSD and Rg data, identified distinct energy minima for all complexes, thereby confirming their thermodynamic stability throughout the simulations (Figs. 8H and J). Collectively, these findings displayed that high MFAP2 expression correlated with enhanced sensitivity to MK-2461 and AZD4547, with MK-2461 exhibiting superior binding affinity, more robust hydrogen bonding interactions, and greater structural stability in complex with MFAP2, supporting its potential as a promising therapeutic candidate for MFAP2-high GC.

### MFAP2 as a multifunctional pan-cancer oncogenic factor

**Fig. 9:**
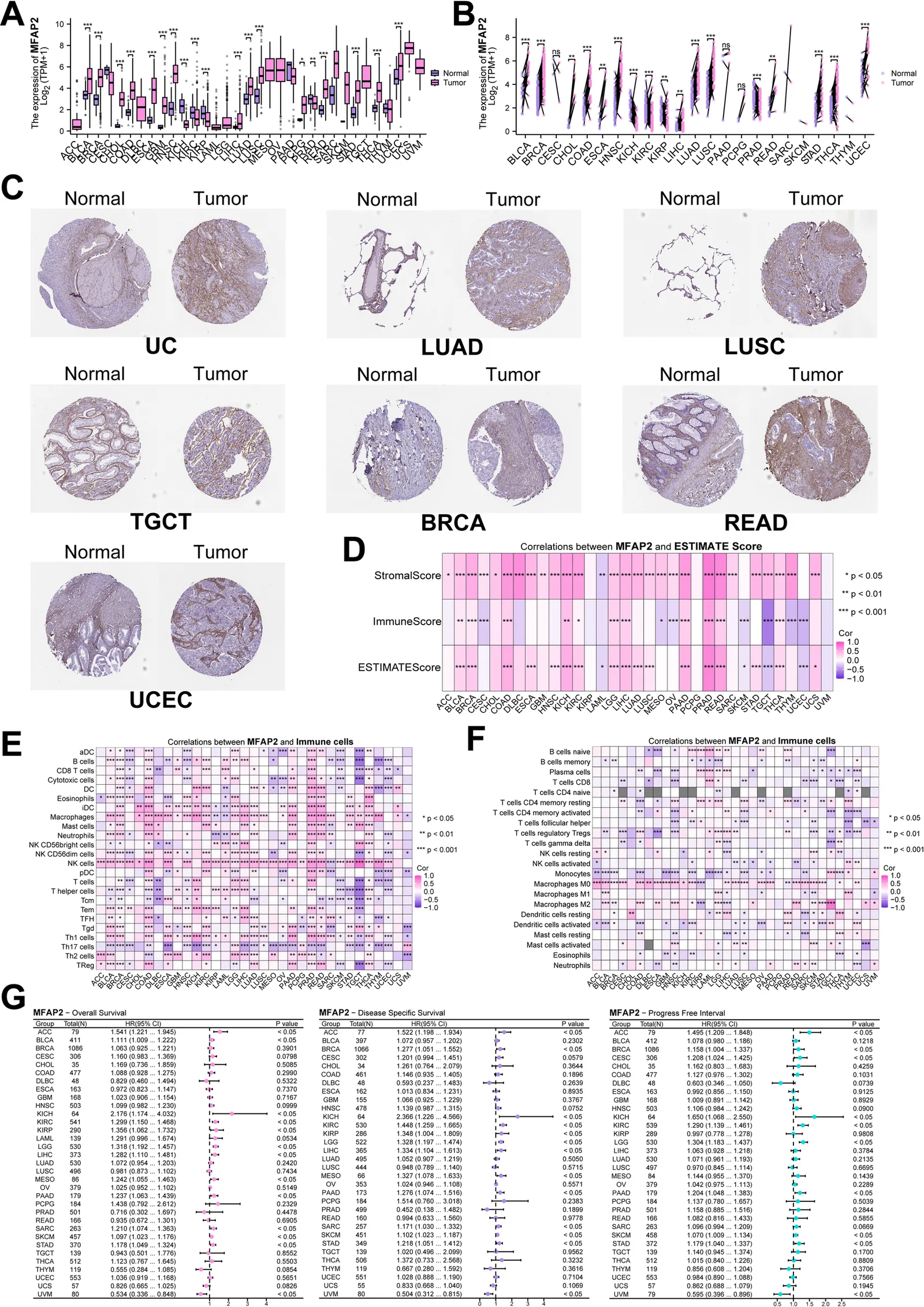
MFAP2 as a multifunctional pan-cancer oncogenic factor. **A** MFAP2 mRNA expression levels in tumors and corresponding normal tissues across various cancer types from TCGA and GTEx datasets. **B** mRNA expression levels of MFAP2 in paired tumor and normal tissues of various cancer types from the CPTAC dataset. **C** Representative IHC images from the HPA database exhibiting MFAP2 protein expression in tumor and normal tissues across multiple cancer types. **D** Heatmap illustrating the correlations between MFAP2 expression and ESTIMATE scores across a pan-cancer landscape. **E-F** Heatmaps presenting the correlation between MFAP2 expression and immune infiltration across multiple cancer types. **G **Forest plots revealing the prognostic value of MFAP2 expression across various cancer types, including OS, DSS, and PFI

To assess the generalizability of our findings in GC, we conducted a systematic pan-cancer analysis of MFAP2. Leveraging TCGA, GTEx, and HPA databases, we evaluated its expression patterns, prognostic value, and impact on the TME. Pan-cancer transcriptomic analysis revealed that MFAP2 was upregulated across most solid tumors, including STAD, UC, LUAD, LUSC, TGCT, BRCA, READ, and UCEC (Fig. 9A). This pattern was robustly confirmed in paired tumor-normal analyses across cancer types (Fig. 9B). Additionally, IHC results from the HPA database further validated the widespread protein-level upregulation of MFAP2 in the cancer types (Fig. 9C). After establishing the ubiquitous expression of MFAP2, we used the ESTIMATE algorithm to investigate its relationship with the TME. MFAP2 expression exhibited a strong positive correlation with stromal and immune scores across nearly all TCGA cancer types, supporting its role as a universal biomarker of stromal activation and immune infiltration (Fig. 9D). Further immune infiltration analysis highlighted a pan-cancer association between MFAP2 and an immunosuppressive phenotype, exhibiting general positive correlation to key immunosuppressive cells, including macrophages, neutrophils, and Tregs (Figs. 9E-F). Additionally, evaluation of OS, DSS, and PFI revealed that high MFAP2 expression was associated with worse prognosis in several cancers, including KIRC, LGG, LIHC, MESO, and STAD (Fig. 9G). In summary, MFAP2 is frequently upregulated across cancers, associated with an immunosuppressive TME, and predicted poor prognosis, highlighting its broad oncogenic role.

## Discussion

Cellular senescence establishes a paradoxical foundation for tumor progression in the TME [25]. Initially serving as a barrier to malignant transformation, sustained SASP ultimately promotes tumorigenesis by fostering a profoundly immunosuppressive niche [26]. This axis associating senescence with immunosuppression is a critical mediator of therapy resistance, particularly to ICIs [27]. However, systematically defining the core effector molecules and their cellular origins within this axis remains a major challenge. To address this, we implemented an integrated multi-omics framework in GC, computationally prioritizing central regulators within the senescence-immunosuppressive network for subsequent functional and clinical validation.

Our integrated analysis first defined SRIS, capturing the co-expression pattern between senescence and immunosuppressive genes. This signature served as a robust prognostic indicator and exhibited the highest activity in CAFs, thereby defining this stromal compartment as the primary cellular niche for the senescence-immunosuppression axis. Notably, this phenomenon is not unique in GC, with convergent evidence emerging from studies in other solid tumors [28, 29]. Using SRIS expression, we further stratified GC into distinct molecular subtypes. The SRIS-high subtype displayed a significant malignant phenotype, characterized by elevated stromal score and poor clinical outcomes, a relationship consistent with the known pro-tumorigenic role of activated stroma in GC [30]. In contrast, although immune score was also elevated in this subtype, the prognosis was nevertheless unfavorable. This discrepancy in the SRIS‑high TME might be related to the enrichment of immunosuppressive populations, such as M2 macrophages and Tregs. The functional dominance of these immunosuppressive subsets likely counteracted the quantitative increase in overall immune infiltration, redirecting the immune response toward immune evasion and adverse prognosis [31]. Interestingly, the SRIS‑high subtype, which was associated with poor clinical prognosis, exhibited elevated expression of canonical cytotoxic markers, including CD8A, CXCL10, GZMA, and GZMB. This observation appears to challenge the conventional notion that increased cytotoxic gene expression uniformly reflects effective antitumor immunity. Notably, this phenomenon is not without precedent. Choo et al. reported that a high density of PD‑1‑positive CD8⁺ T cells in the GC correlated with poorer OS [32]. Similarly, Katayama et al. presented that GC patients with high CD8⁺ T cell infiltration exhibited an unfavorable prognosis, accompanied by upregulated chemokines and CTLA‑4 expression [33]. These findings were in line with our data, which revealed that the SRIS‑high subtype was associated with concurrent upregulation of multiple chemokines and immune checkpoint molecules. Collectively, these observations suggested that the elevated CD8⁺ T cells within the SRIS‑high TME may represent a functionally exhausted population, which failed to mount an effective antitumor response and ultimately contributed to adverse clinical outcomes. Additionally, our subtyping analysis only based on gene expression profiles, which may not fully capture the inherent complexity of the TME. The application of more advanced heuristic or chaotic optimization clustering algorithms in future studies could further refine patient stratification and reveal subtle subgroup differences of potential biological and therapeutic significance [34].

Recent advances in optimized machine learning frameworks have shown great potential in improving predictive accuracy and handling complex datasets [35]. Employing an integrative machine learning framework, we identified MFAP2 as the central prognostic regulator of SRIGs in GC, which was further validated using multiple clinical cohorts, RT-qPCR, and IHC. MFAP2, a key secreted component of extracellular microfibrils also designated as MAGP1, is integral to the structure and homeostasis of the mammalian ECM [36]. In humans, MFAP2 expression is enriched in stromal cells, particularly fibroblasts, and in endothelial and smooth muscle cells, reflecting its central role in ECM production and remodeling [37]. Functionally, MFAP2 interacts with core ECM components, including fibrillin-1, tropoelastin, and fibronectin, thereby bridging structural integrity with cellular signaling within the tissue microenvironment [38, 39]. Beyond its physiological roles, MFAP2 is implicated in oncogenesis. Its overexpression in multiple solid tumors drives pro-tumorigenic processes, including EMT, invasion, and metastasis, frequently via dysregulation of pathways, including TGF-β and Wnt/β-catenin [40]. Consistent with prior evidence, elevated MFAP2 expression in GC was significantly associated with clinicopathological markers of tumor aggressiveness and worse prognosis [41]. Functional enrichment analysis revealed that samples with high MFAP2 expression were significantly enriched in pathways associated with tumorigenesis, progression, and ECM reprogramming, such as TGF-β signaling and ECM receptor interaction pathways.

By integrating bulk RNA-seq and scRNA-seq data, we identified and characterized a functionally distinct MFAP2⁺ eCAFs subset that was markedly expanded in tumors, whose abundance directly correlated with poor OS. Pseudotime analysis identified MFAP2 as a critical molecular node driving the phenotypic transition of normal fibroblasts into pro-tumorigenic eCAFs, and this differentiation trajectory was characterized by robust activation of ECM-remodeling genes. Consistently, spatial omics analysis further validated the strong co-localization of MFAP2 with key ECM-remodeling genes, typified by POSTN [42]. Collectively, this expression signature suggests that MFAP2⁺ eCAFs may orchestrate the formation of a specialized tumor stromal niche through ECM and stromal architecture remodeling, which could help establish a permissive microenvironment conducive to GC cell proliferation, invasion, and maintenance of tumor structural integrity.

A compelling finding of our study is the distinct spatial and functional crosstalk between MFAP2⁺ eCAFs and M2 macrophages. Spatial transcriptomics and mIF revealed frequent co-localization of MFAP2⁺ eCAFs with M2 macrophages in the tumor stroma, supporting the possibility that MFAP2⁺ eCAFs may function as a central signaling hub in the TME. Cell-cell communication analysis further verified that MFAP2⁺ eCAFs established specific interactions with macrophages via CSF1-CSF1R and C3-C3AR1 signaling axes, revealing a key mechanism through which MFAP2 could potentially modulate the immunosuppressive TME [43]. CSF1-CSF1R axis represents a canonical pathway governing macrophage proliferation, differentiation, and polarization. Our data indicate that MFAP2⁺ eCAFs secrete CSF1, which binds to CSF1R on macrophages and might activate downstream PI3K, ERK1/2, and JNK cascades, potentially driving macrophage polarization toward the pro-tumorigenic M2 phenotype [44, 45]. Consistent with this in silico and spatial inference, our in vitro THP-1 macrophage overexpression assays offered supplementary functional evidence: enforced MFAP2 upregulation significantly elevated transcripts of the pan-macrophage marker CD68, anti-inflammatory cytokine IL-10, and prototypical M2 surface markers MRC1 and CD163, further implying that MFAP2 is capable of skewing macrophages toward an anti-inflammatory, M2-like transcriptional profile. Complement activation has emerged as a critical regulator of tumor progression. Accumulating evidence reveals that C3 activation in the TME generates functional fragments that bind to macrophage receptors, modulating macrophage activation and promoting M2 polarization [46, 47]. In agreement with these reports, Li Y et al. demonstrated that the C3-C3AR1 axis mediates robust crosstalk between CAFs and macrophages to form a pro-tumorigenic stromal-myeloid niche, and blockade of this axis markedly enhances the efficacy of immune checkpoint blockade [8]. These two pathways may act synergistically to facilitate macrophage recruitment and M2 polarization. M2 macrophages secrete immunosuppressive cytokines, including IL-10 and TGF-β, which suppress cytotoxic T lymphocyte activity, promote Tregs expansion, and impair natural killer cell function via dysregulated antigen presentation, which is likely to gradually establish and maintain a local immunosuppressive microenvironment [48]. This mechanism aligns with our previous observation that MFAP2-high tumors exhibit increased immunosuppressive cell infiltration and resistance to ICIs. Consequently, MFAP2⁺ eCAF-mediated crosstalk through these two signaling axes could represent one critical molecular link connecting stromal remodeling to tumor immune evasion. Our findings also provide a novel therapeutic rationale: Targeting CSF1-CSF1R or C3-C3AR1 axis to disrupt abnormal MFAP2⁺ eCAF–macrophage communication might help reverse the immunosuppressive TME and could potentially improve the clinical efficacy of ICB therapy in patients with MFAP2-high GC.

The TME is increasingly recognized as a critical element of immunotherapy efficacy. Among its various components, CAFs play a important role in driving immune evasion and resistance to ICIs [49, 50]. As a CAF-derived mediator, MFAP2 may play an important role in this process. Therefore, we further evaluated the clinical value of MFAP2 in ICIs. Our analyses across pan-cancer cohorts established MFAP2 as a robust prognostic indicator and a predictive biomarker for immunotherapy response. In patients with pan-cancer treated with ICIs, MFAP2 expression was significantly higher in non‑responders than in responders, and elevated MFAP2 expression was tightly correlated with inferior PFS and OS. Receiver operating characteristic (ROC) curve analyses further demonstrated consistent and robust predictive performance across multiple independent pan‑cancer cohorts. These findings were further supported by the GC cohort, in which we validated the expression pattern of MFAP2, consolidating its potential as a reliable biomarker for patient stratification in ICI therapy.

To tackle ICI resistance in MFAP2-high tumors, our drug sensitivity screening identified AZD4547 and MK-2461 as promising therapeutic agents. AZD4547 is a selective inhibitor of the FGFR family, which directly abrogates FGFR-driven signaling cascades [51]. Preclinical studies have demonstrated that AZD4547 elicits robust antitumor activity in GC models harboring FGFR2 amplification [52]. Given that MFAP2-high tumors are characterized by pronounced ECM remodeling and CAF activation, processes frequently regulated by the FGFR axis, we postulate that the FGFR pathway is aberrantly activated in this tumor niche, rendering malignant cells reliant on this signaling cascade for sustained oncogenicity [53]. Similarly, MK-2461, a MET inhibitor that targets MET/HGF-driven invasion and EMT, suppresses downstream signaling via inhibiting FGFR phosphorylation [54]. This result implies that the susceptibility of MFAP2‑high tumors to MK‑2461 may involve inhibition of the FGFR pathway.

Interestingly, our molecular docking results showed the possibility that AZD4547 and MK‑2461 may directly bind to MFAP2, an ECM structural protein rather than a conventional kinase target. Molecular dynamics simulations further confirmed the conformational stability of these complexes throughout the simulation period. This finding warranted critical consideration. On one hand, an ECM protein could modulate drug response through an extracellular “sponge” effect. Under the circumstances, the protein blocked the drug and reduced its bioavailability to target cells, potentially contributing to therapeutic resistance. However, this possibility appeared inconsistent with our drug sensitivity data. Our data showed a negative correlation between MFAP2 expression and IC50 values, indicating that MFAP2‑high tumors were preferentially sensitive to these agents rather than resistant. On the other hand, the docking results did suggest a possible direct interaction between these kinase inhibitors and MFAP2. Whether this binding has any functional consequences remains to be determined. The biological significance of this docking observation therefore requires experimental validation.

The significance of our findings extends beyond GC. Our pan-cancer characterization demonstrates that MFAP2 constitutes a universal hallmark of the immunosuppressive TME across multiple human malignancies. The consistent upregulation of MFAP2, together with its robust association with elevated stromal scores, M2 macrophage infiltration, and adverse prognosis across diverse solid tumors (lung, breast, and colon cancers), suggests that MFAP2-driven stromal-immune regulatory axis represents a fundamental feature of solid tumor progression. This pan-cancer conservation further implies that MFAP2 may serve as a broad-spectrum therapeutic target, and strategies targeting the MFAP2-mediated tumor niche could benefit patients with GC and be extrapolated to other “cold” tumors characterized by dense stromal deposition and myeloid cell-driven immune exclusion. Notably, MFAP2 exhibited consistent pro-tumorigenic associations in cancer contexts. However, prior studies in non-digestive fibrotic tissues have reported different roles, which indicated that MFAP2 functions were context-dependent [55]. These discrepant observations may arise from tissue-specific microenvironmental differences, distinct CAF subpopulations, or the opposing roles of MFAP2 in homeostatic maintenance versus pathological remodeling. Such context-dependent functional heterogeneity warrants further investigation.

Collectively, by identifying MFAP2 as a critical molecular bridge associating senescent CAFs to immunosuppressive macrophages, our study provides a novel mechanistic rationale for patient stratification and targeted drug discovery. Future prospective clinical trials with larger patient cohorts are warranted to validate the utility of MFAP2 in selecting candidates for AZD4547 or MK-2461 therapy, potentially paving the way for more effective and individualized combination strategies in the era of precision oncology.

## Conclusion

In this study, we identified MFAP2 as the hub SRIG in GC, with its expression predominantly restricted to eCAFs. Our findings suggest that MFAP2 may contribute to pro-tumorigenic fibroblast differentiation and ECM remodeling. Through potential stromal-immune crosstalk involving CSF1-CSF1R and C3-C3AR1 axes, MFAP2 was associated with M2-like macrophage polarization, immune evasion, and unfavorable immunotherapy responses. These observations highlighted MFAP2 as a candidate prognostic biomarker and a potential therapeutic target for GC, warranting further functional and clinical investigation to inform precision immunotherapy strategies.

## Supplementary Information

Supplementary Material 1.

Supplementary Material 2.

## Data Availability

The datasets generated and/or analyzed during the current study are available in the GEO repository (http://www.exorbase.org/), TCGA repository (http://cancergenome.nih.gov/, BEST repository (https://rookieutopia.hiplot.com.cn/app_direct/BEST/), and CPTAC repository (https://cprosite.ccr.cancer.gov/).

## Abbreviations

GC: Gastric cancer
ICI: Immune checkpoint inhibitor
SRIGs: Senescence-related immunosuppressive genes
TME: Tumor microenvironment
MFAP2: Microfibril-associated protein 2
eCAFs: Extracellular matrix cancer-associated fibroblasts
ECM: Extracellular matrix
OS: Overall survival
SASP: Senescence-associated secretory phenotype
scRNA-seq: Single-cell RNA sequencing
WGCNA: Weighted gene co-expression network analysis
TCGA: The Cancer Genome Atlas
GEO: Gene Expression Omnibus
FDR: False discovery rate
DEGs: Differentially expressed genes
TIDE: Tumor Immune Dysfunction and Exclusion
GO: Gene ontology
KEGG: Kyoto encyclopedia of genes and genomes
GSEA: Gene set enrichment analysis
Kaplan–Meier: K-M
TIME: Tumor immune microenvironment
ssGSEA: Single-sample gene set enrichment analysis
KNN: K-nearest neighbor
SASA: Solvent accessible surface area
mIF: Multiplex immunofluorescence
RT-qPCR: Real-time polymerase chain reaction
SRIS: Senescence-related immunosuppressive signature
PCA: Principal component analysis
EMT: Epithelial-mesenchymal transition
HPA: Human Protein Atlas
TGF-β: Tumor growth factor-β
apCAFs: Antigen-presenting CAFs
iCAFs: Inflammatory CAFs
MFAP2⁺ eCAFs: MFAP2-positive eCAFs
OE: Overexpression
PFS: Progression-free survival
Rg: Radius of gyration
