# Supplementary material for: Senescence-related immunosuppressive gene MFAP2 orchestrates stromal-immune crosstalk and immunotherapy resistance in gastric cancer
Source: BMC Cancer. 2026 Aug 4;26:1337. doi: 10.1186/s12885-026-16660-5 (PMC13726166; doi:10.1186/s12885-026-16660-5)
Supplement: Supplementary file 1 [file 12885_2026_16660_MOESM1_ESM.docx]

**Supporting Information**

**Fig.S1** Quality control and preliminary analysis of single-cell RNA sequencing (scRNA-seq) data. **A-F** Quality control metrics for scRNA-seq data. **G** Cell filtering scatter plot. **H-M** Dimensionality reduction visualization of scRNA-seq data.

**Fig.S2** Unsupervised cluster analysis and differential expression of functional gene sets. **A** Delta area plot shows the relative change in CDF area to determine the optimal number of clusters (k)**. B** Tracking plot visualizes cluster assignment stability across resampling iterations. **C** Consensus CDF curves assess cluster separation for different k values, with steeper curves indicating better clustering performance. **D-H** Box plots revealed the expression of pro-inflammatory chemokines/cytokines, EMT-related genes, HLA antigen presentation genes, chemokine receptors, and immune regulatory cytokines between cluster C1 and cluster C2.

**Fig.S3** Identification of critical prognostic SRIG based on machine learning algorithms. **A-G** The remaining eight machine learning algorithms (AdaBoost, CART, DT, GBM, MLP, NN and RF) used for identifying critical prognostic SRIG. **H-K** Real-time PCR detection of GPX8, SERPINE1, LBH and P4HA3 of clinical samples.

**Fig.S4** Expression, clinical relevance, and functional enrichment analysis of MFAP2. **A-B** Violin plots showed MFAP2 expression stratified by tumor stage, N stage and M stage. **C** Kaplan-Meier survival curves showed PFS, DFS and DSS stratified by high and low MFAP2 expression. **D** Violin plots displayed MFAP2 expression in different molecular subtypes (Invasive, Metabolic, Proliferative, Unstable). **E** GSEA Hallmark pathway enrichment plot showed significantly enriched biological pathways in the MFAP2-high expression group.

**Fig.S5** Quality control and preliminary spatial transcriptomics analysis of two independent tissue sections. **A-B** For each sample, analysis started with a hematoxylin and eosin (H&E) stained image for histopathological context, followed by spatial transcriptomics quality control metrics, and concludes with visualizations.

**Fig.S6 Dimensionality reduction visualization and fibroblast subpopulations of integrated scRNA-seq data.**

**A-B** T-SNE and UMAP dimensionality reduction plots colored by project origin. **C** UMAP dimensionality reduction plots colored by sample ID. **D-E** T-SNE and UMAP dimensionality reduction plots colored by cluster assignment. **F** t-SNE plot annotated by cell type, showing the spatial distribution of fibroblast subpopulations.
