# Supplementary material for: Senescence-related immunosuppressive gene MFAP2 orchestrates stromal-immune crosstalk and immunotherapy resistance in gastric cancer
Source: BMC Cancer. 2026 Aug 4;26:1337. doi: 10.1186/s12885-026-16660-5 (PMC13726166; doi:10.1186/s12885-026-16660-5)
Supplement: Supplementary file 2 [file 12885_2026_16660_MOESM2_ESM.docx]

| **Characteristics** | **Classification** | **Number of patients (n)** |
| --- | --- | --- |
| Age | ≤60 years | 3 |
|  | >60 years | 2 |
| Gender | Male | 4 |
|  | Female | 1 |
| AJCC TNM stage | I–II | 2 |
|  | III–IV | 3 |

**Supplementary Table S1**. Clinicopathological characteristics of the GC validation cohort (n = 5).
